# The Use of Porous Silica Particles as Carriers for a Smart Delivery of Antimicrobial Essential Oils in Food Applications

**DOI:** 10.1021/acsomega.1c03549

**Published:** 2021-11-03

**Authors:** David J. Sullivan, Tom F. O’Mahony, Malco C. Cruz-Romero, Enda Cummins, Joseph P. Kerry, Michael A. Morris

**Affiliations:** † AMBER Research Centre and the School of Chemistry, Trinity College Dublin, Dublin 2, Ireland; ‡ Food Packaging Group, School of Food & Nutritional Sciences, University College Cork, Cork T12 K8AF, Ireland; § UCD School of Biosystems and Food Engineering, Agriculture and Food Science Centre, University College Dublin, Belfield, Dublin 4, Ireland

## Abstract

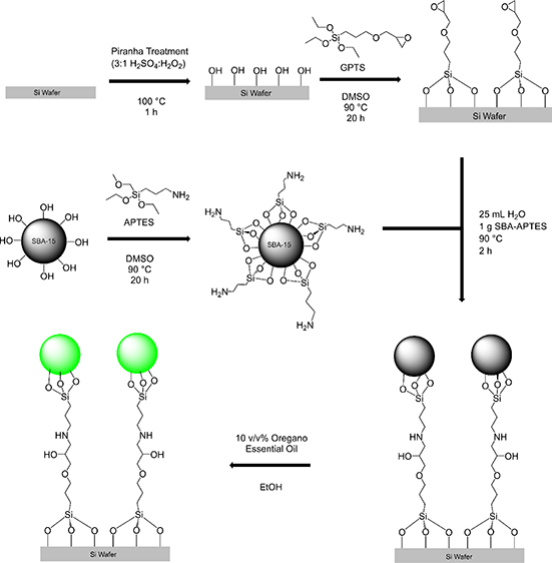

The objective of this study was to design, develop, and quantify the effectiveness of a simple method to facilitate the smart delivery of antimicrobial essential oils (EOs) *via* their absorption into a chemically bound high surface area support material. To this end, Santa Barbara Amorphous 15 (SBA-15) was functionalized by means of a post-synthetic reaction using (3-aminopropyl)triethoxysilane (APTES) to create an amine-terminated SBA-15 (SBA-APTES), and functionalization was confirmed by FTIR, TGA, and N_2_ isotherm analysis. Amine-modified SBA-15 was then grafted to a 3-glycidyloxypropyltrimethoxysilane (GPTS)-modified silicon (Si) surface (Si-GPTS), and subsequent attachment to the GPTS-modified surface was confirmed through XPS, dynamic contact angle, and SEM analysis. The smart delivery devices (SBA-15 and SBA-APTES) were then loaded with antimicrobial oregano essential oil (OEO) and the antimicrobial activity was assessed against common food spoilage microorganisms *Escherichia coli*, *Bacillus cereus*, *Staphylococcus aureus*, and *Pseudomonas fluorescens*. Antimicrobial activity results indicate that both SBA-OEO and SBA-APTES-OEO have good antimicrobial activity and that functionalization of bare SBA-15 with APTES has no effect on antimicrobial activity (*P* > 0.05) compared to SBA-OEO. Moreover, it appears that direct surface coating of the modified SBA to a surface substrate may not provide a significant quantity of oil needed to elicit an antimicrobial response. Nevertheless, given the strong absorption properties of SBA materials, good antimicrobial activity, and the GRAS nature of SBA-OEO and SBA-APTES-OEO, the results found in this study open potential applications of the functionalized carrier materials.

## 1 Introduction

Global food security challenges are of increasing concern due to a multitude of factors such as an increasing global population (estimated to reach 9 billion by 2050), increasing rural-to-urban migration, and climate change, and these are putting increased pressure on global food production and supply chains.^1^ To alleviate the pressure from these challenges and due to the associated “health risks” associated with metal ion-based antimicrobials, extensive research has been carried out on the incorporation of natural antimicrobial materials (NAMs), i.e., materials derived from a naturally occurring source such as plants, animals, etc.^1^ Essential oils (EOs) are defined as a product obtained by steam distillation from a natural raw material of plant origin by the International Organization for Standardization (ISO) (2013). These materials have favorable properties for use in food contact applications such as good antimicrobial activity and GRAS (Generally Recognized as Safe) status approved by the Food and Drug Administration (FDA) and are acceptable to consumers from their historical use as natural flavorings.^2,3^ EOs are mixtures of secondary metabolite compounds such as terpenes, terpenoids, and phenylpropanoids. Specifically, metabolites found in EOs such as *p*-cymene, thymol, and eugenol can synergistically act together to contribute to the antimicrobial activity through interactions with cell wall components.^4,5^

Despite EOs having good antimicrobial properties, roadblocks in their application persist as they are hydrophobic, thermolabile, and photosensitive and can impart a strong effect on organoleptic properties.^1^ Moreover, the volatile nature of EOs means that they are highly susceptible to autoxidation, isomerization, and thermal rearrangements.^6−8^ To overcome these limitations, several strategies such as EO incorporation into sachets and edible films have been used. However, these approaches also present their own technical problems including impacts on the organoleptic properties of food and limited suitability to certain packaging systems.^9^ One solution to overcome these roadblocks is the encapsulation of EOs into a porous and mesoporous siliceous material,^10^ and examples include Santa Barbara Amorphous (SBA-15) or Mobil Composition of Matter No. 41 (MCM-41). These materials can protect EOs from environmental stressors and allow for controlled release of EOs^11^ while also facilitating targeted release of EOs onto the food surface, where most of the spoilage occurs. In particular, the use of SBA-15 as an encapsulator is attractive due to its current use in the food sector as a catalyst in the synthesis of nutrients, bioactive molecules, and sensor technology and as a carrier to design smart delivery systems.^11^ SBA-15 materials have greater mechanical and hydrothermal stability over other similar siliceous materials such as MCM-41.^12,13^ In addition, SBA-15 has adjustable nanopore size,^10^ ordered pore structure,^14^ large specific surface area (∼1000 m^2^ g^–1^),^14^ and relatively large void volume.^12^ Furthermore, SBA-15 materials are also considered GRAS and are an authorized additive in the European Union (E-551).^11^ SBA-15 can also be readily functionalized with various organic functional-containing groups such as 3-aminopropyltriethoxysilane (APTES), which are covalently grafted onto the surface of the porous silica structure *via* hydrolysis and/or condensation reaction mechanisms. This results in an amine-modified porous silica that retains the mesoporous silica’s favorable physical properties^15^ and introduces an amine group that can be used as an intermediate for further functionalization with other organo-functional alkoxysilanes such as 3-glycidoxypropyltrimethoxysilane (GPTS). The epoxy group on GPTS can readily undergo poly-addition to the amine group or hydrolytic ring opening. In addition, the trialkoxysilyl moiety of GPTS can undergo hydrolysis and condensation reactions with terminal −OH groups.^16^ This method of attachment could be used to graft functionalized SBA to food packaging surfaces and be suitable for food applications as the covalent attachment of APTES and GPTS can be carried out in deionized water. This anchors the SBA support material (pre- or post-loaded with EOs) to a packaging surface with potential long-term antimicrobial properties due to the support material.^17^

Mesoporous silica supports for EO delivery in food applications have been reported elsewhere, and Park et al.^18^ found that MCM-41 and SBA-15 loaded with natural antimicrobial allyl isothiocyanate were antimicrobially active against *Escherichia coli*, *Bacillus cereus*, and *Pichia anomola.* A study by Ruiz-Rico et al.^11^ reported a significant reduction in the concentration of *Listeria innocua* in pasteurized skimmed milk using vanillin grafted onto the surface of MCM-41.

Nonetheless, to the best of our knowledge, no studies have investigated the novel approach of using amine-functionalized SBA-15 grafted to a GPTS-modified surface as a support material for EOs. This would allow slow release of naturally occurring biocides such as EOs and enhance the antimicrobial effect while also overcoming challenges such as their cost, taste/smell, and effects on polymer packaging materials. Therefore, the aim of this study was to identify a method to covalently attach amine-functionalized SBA-15 (SBA-APTES) to a GPTS-modified Si surface to act as a support material for OEO. The synthesized materials were subsequently characterized, and their antimicrobial activity was assessed.

## 2 Results and Discussion

### 2.1 Functionalization of Bare SBA-15 with 3-Aminopropyltriethoxysilane

**Figure 1:**
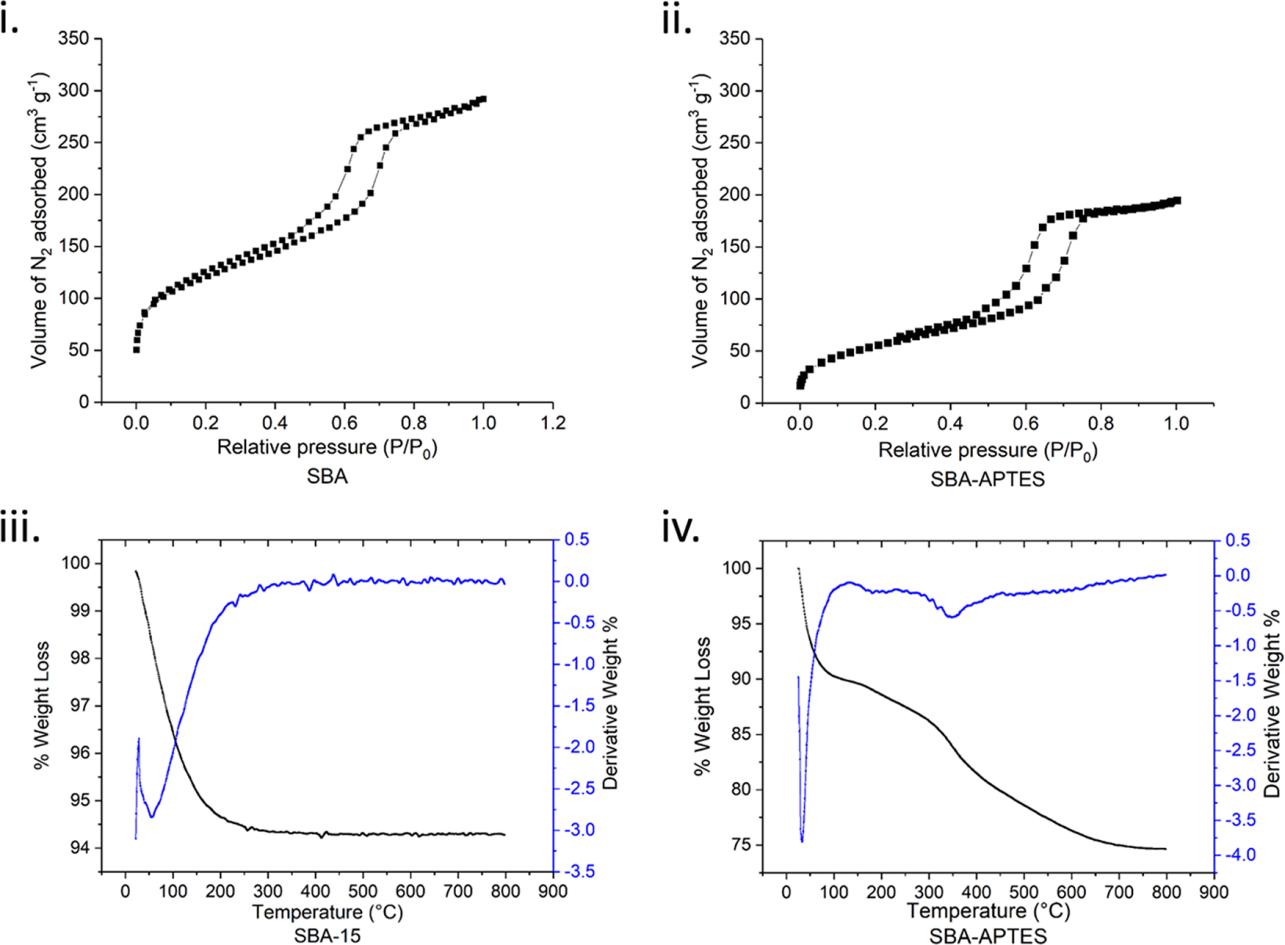
Nitrogen adsorption/desorption isotherms at 77 K for (i) SBA-15 and (ii) SBA-APTES and TGA curves and the first derivative of (iii) bare SBA-15 and (iv) SBA-APTES.

The functionalization of SBA-15 with APTES was assessed using N_2_ adsorption/desorption isotherms, TGA, TEM, and FTIR. To this end, the N_2_ adsorption/desorption isotherms of bare SBA-15 and SBA-APTES at 77 K are shown in Figure 1i,ii, while the textural properties including surface area determined by the BET method (*S*_BET_), BJH pore size (*D*_BJH_), and total pore volume (*V*_total_) are shown in Table 1. Bare SBA-15 and SBA-APTES both show a type IV isotherm with H1 hysteresis and a sharp increase in adsorbed volume, which is a reported characteristic of a highly ordered mesoporous material.^14^ However, the amount of N_2_ absorbed was reduced after SBA-15 was grafted with APTES, which may be due to the space within the pores being filled in with the APTES molecule.^15^ Textural properties calculated from the N_2_ adsorption/desorption isotherms of SBA-15 and SBA-APTES show that the *S*_BET_ values for pure SBA-15 and SBA-APTES were 436.33 and 200.60 m^2^ g^–1^, respectively. This indicates that the surface area of SBA-APTES was lower than SBA-15 and this result agreed with Hernández-Morales et al.^12^ The pore size distribution curves of bare SBA-15 and SBA-APTES were calculated from the N_2_ adsorption/desorption isotherms using the BJH model and were estimated from the peak positions of the BJH pore size distribution curves measured from both the adsorption and desorption isotherms. The pore sizes of SBA-15 and SBA-APTES were 55 and 54 Å, respectively, and were in good agreement with results reported by Maria Chong and Zhao.^14^ The pore volume of SBA-15 was 0.452 cc g^–1^ and that of SBA-APTES was 0.301 cc g^–1^, indicating that functionalization occurred on the surface and in the pores of SBA-15 as evidenced by the large reduction in pore volume and was in agreement with the literature on silica functionalization.^12,14,19^

**Table 1:** BET Specific Surface Area (*S*_BET_), BJH Pore Size (*D*_BJH_), and Total Pore Volume (*V*_p_) Properties of Bare SBA-15 and SBA-APTES

	*S*_BET_ (m^2^ g^–1^)	*V*_p_ (cc g^–1^)	*D*_BJH_ (Å)
SBA	436.33	0.452	55
SBA-APTES	200.60	0.301	54

Thermogravimetric analysis (TGA) of bare SBA-15 and SBA-APTES showing the weight loss curves is shown in Figure 1iii,iv. An initial weight loss of 4.8% observed in SBA-15 was associated with the removal of physisorbed water (Figure 1iii). A further weight loss of 0.8% is due to the removal of chemisorbed water. The final weight loss of 0.1% was attributed to surface silanol groups decomposing to release water and, subsequently, the formation of silane bridges on the SBA surface. Likewise, TGA analysis of SBA-APTES shows an initial weight loss of 10.4% associated with the removal of physisorbed water, while a further 3.5% was from the removal of chemisorbed water on the SBA-APTES surface (Figure 1iv). In addition, the APTES decomposition can been seen to occur typically in the 300–400 °C range and accounted for an overall weight loss of 4.65%.^12,20^ The final weight loss was associated with the dehydroxylation by condensation of silanols on the surface of the SBA-APTES.^14^ The larger weight loss observed with respect to bare SBA-15 has been attributed to the presence of the amino groups. Those groups have high thermal stability (above 250 °C), suggesting that the bare SBA-15 silica sample has a stabilizing effect on the temperature of decomposition of the surface species.^12^

**Figure 2:**
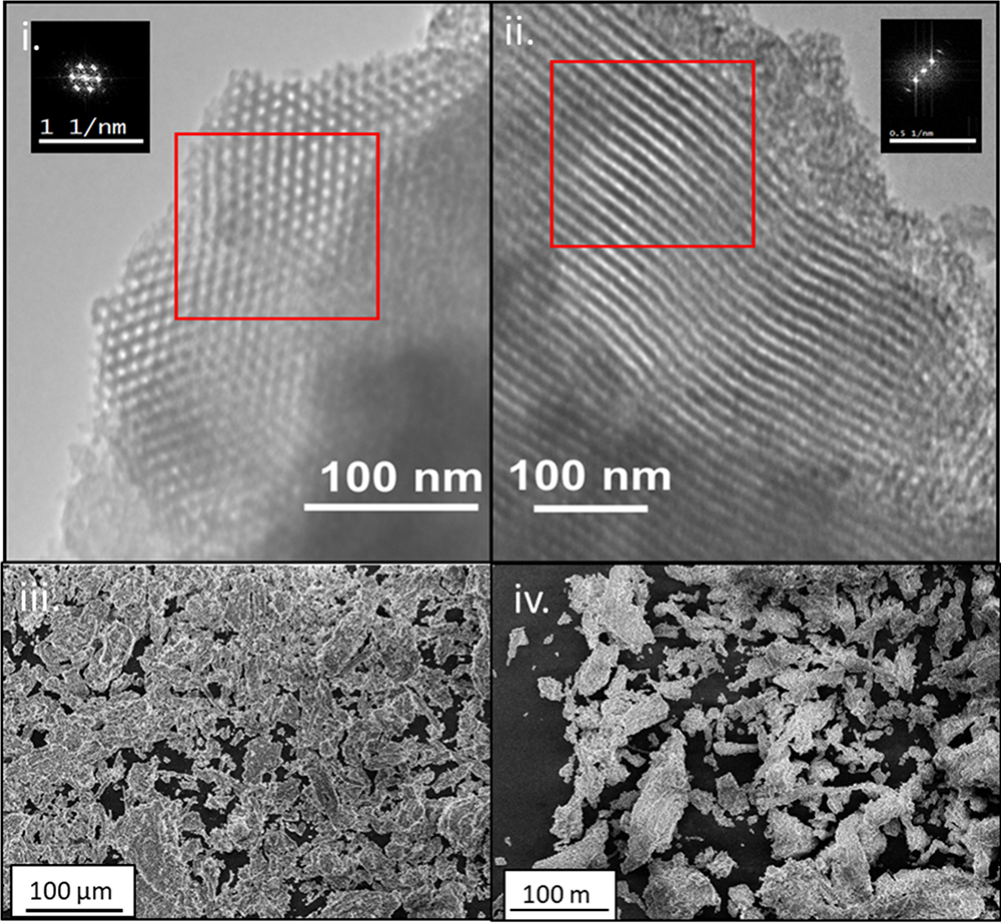
TEM images of SBA-15 showing (i) a well-ordered hexagonal array of mesopores and (ii) parallel nanotubular pores, with the insets showing the FFT analysis and SEM images of (iii) SBA-15 and (iv) SBA-APTES.

The structure of bare SBA-15 was also analyzed using transmission electron microscopy (TEM) (Figure 2). TEM analysis shows a well-ordered hexagonal array structure (Figure 2i) with nanotubular pores (Figure 2ii), which are typical of SBA materials and have been widely reported.^12^ These results were further confirmed by using fast Fourier transform (FFT) analysis, confirming that the crystal lattice of SBA-15 has a well-ordered hexagonal array structure with nanotubular pores (Figure 2i,ii (inset)). Scanning electron microscopy (SEM) analysis of SBA-15 and SBA-APTES is also shown in Figure 2iii,iv.^21^

**Figure 3:**
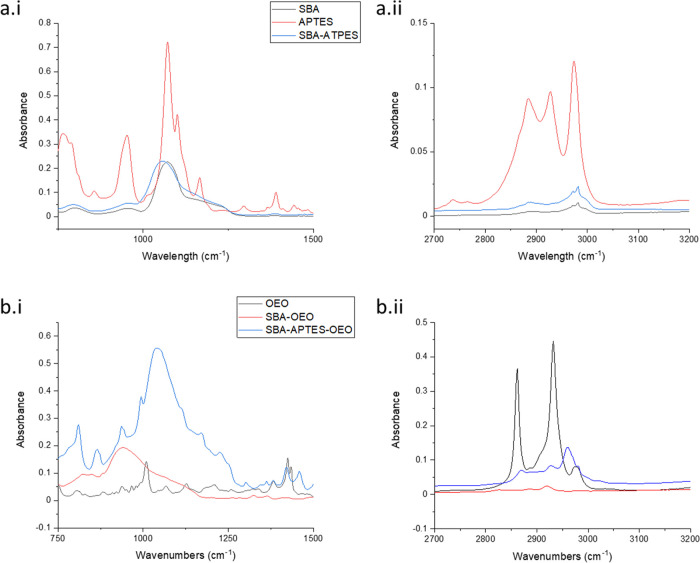
(a) FTIR spectra of SBA (black line), APTES (red line), and SBA-APTES (blue line) between 750–1500 cm^–1^ (a, i) and 2700–3200 cm^–1^ (a, ii) and (b) FTIR spectra (ii) of OEO (black line), SBA-OEO (red line), and SBA-APTES-OEO (blue line) between 750–1500 cm^–1^ (b, i) and 2700–3200 cm^–1^ (b, ii).

The FTIR spectra of bare SBA-15, APTES, and SBA-APTES are shown in Figure 3a. The presence of the peaks between 1000 and 1130 cm^–1^ in bare SBA-15 indicates the symmetrical and asymmetrical stretching of the Si–O–Si backbone of SBA,^14^ the peak at 3400 cm^–1^ indicates the presence of silanol groups that cover the surface of SBA and are cross hydrogen-bonding with adsorbed water,^15^ and the peak at 3740 cm^–1^ corresponds to the symmetric stretching of terminal Si–O–H. With respect to APTES, the FTIR spectra show characteristic peaks at 1000–1130 cm^–1^, which are characteristic of symmetrical and asymmetrical stretching of Si–O groups. The peak at 1388 cm^–1^ is attributed to a stretching C–N bond and peaks at 2884, 2962, and 2974 cm^–1^ are attributed to stretching C–H bonds. Moreover, a C–O terminal was observed at 1070 and 1600 cm^–1^ due to the H bending on the N of the amine group. Several new peaks on the grafted SBA-APTES compared to bare SBA-15 were observed due the presence of APTES. A greater intensity of the peaks at 2927 and 2857 cm^–1^ was observed due to vibrational stretching C–H groups from APTES, and a peak at 1646 cm^–1^ was due to the H bending on the N of NH_2_. When SBA-ATPES was compared to the spectra of SBA and ATPES, the peak characteristics of both SBA and APTES were observed. Furthermore, the disappearance of the terminal Si–OH stretch at 3740 cm^–1^ would suggest that the ethoxy group from ATPES has bound to the surface of SBA.

Overall, these results indicate that APTES has been grafted onto the surface of SBA-15 and are in agreement with results reported in the literature.^22−24^ The proposed mechanism of SBA-15 functionalization with APTES was through the condensation and hydrolysis of the terminal Si–OH from SBA-15 with the alkoxy group (−OCH_2_CH_3_) of ATPES, releasing H_2_O and forming a stable covalent bond between SBA and APTES as previously reported.^23,25,26^

### 2.2 Attachment of GPTS to Si Coupons to Develop Si-GPTS-APTES-SBA Materials

**Figure 4:**
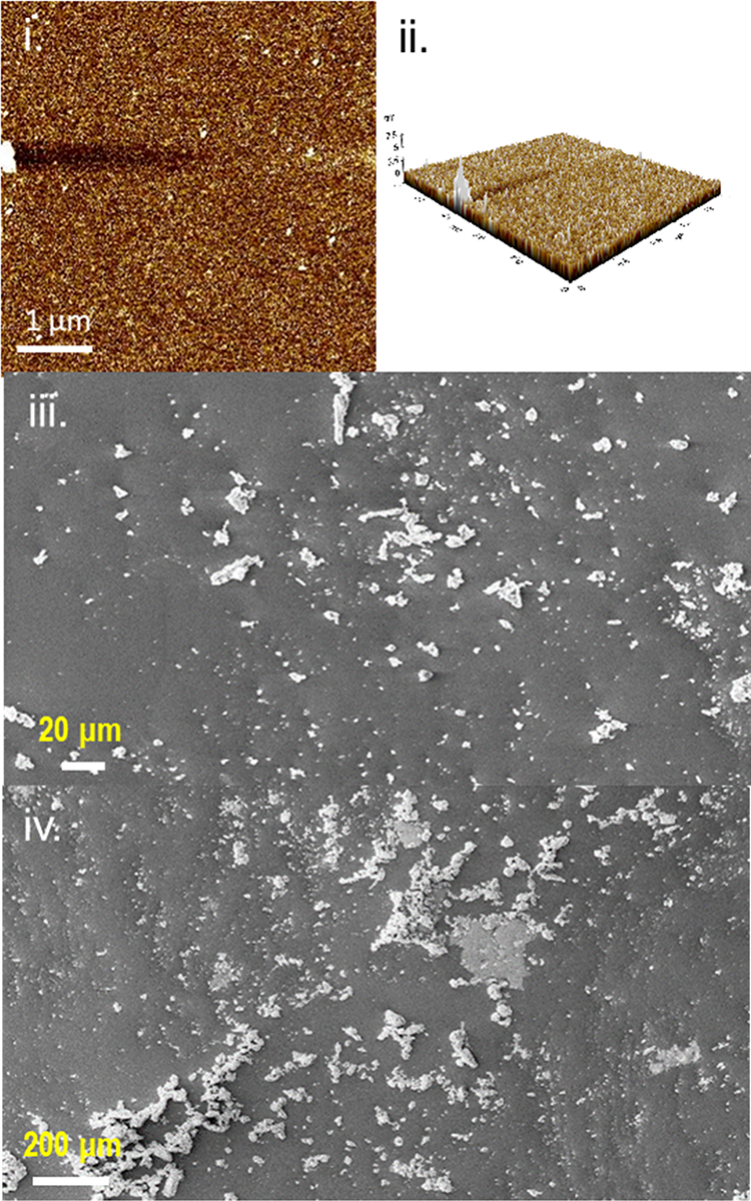
AFM images of Si-GPTS showing (i) 2D and (ii) 3D topographical images and SEM images of the Si-GPTS-APTES-SBA surface at (iii) 200 μm and (iv) 20 μm.

Dynamic contact angle (DCA) measurements of Si–OH (piranha-treated Si), Si-GPTS, and Si-GPTS-APTES-SBA are shown in Table 2. For piranha-treated Si wafers (Si–OH), the wettability was found to be 9°; however, after functionalization of Si–OH with GPTS, the wettability decreased to 57° from the substitution of the hydrophilic hydroxyl sites with the hydrophobic GPTS.^22^ Following the functionalization of Si-GPTS with SBA-APTES, the wettability was found to increase again with respect to Si-GPTS to 26° due to the presence of the hydrophilic APTES on the surface. In addition, the DCAs of Si-GPTS and Si-GPTS-APTES-SBA were also measured using diiodomethane as the dispersive solvent, and results showed contact angles of 40° and 26° for Si-GPTS and Si-GPTS-APTES-SBA, respectively. The surface free energies (SFEs) for Si-GPTS and Si-GPTS-APTES-SBA were determined using the Owens–Wendt model (eq 1) and were found to be 49 and 67 mJ m^–2^, respectively. The change in the SFE would suggest that SBA-APTES has attached to the Si-GPTS substrate. Furthermore, the topographical features of Si-GPTS were measured using AFM analysis (Figure 4i). AFM analysis showed that Si-GPTS has a smooth topographical surface with a surface roughness (Ra) of 0.22 nm and small agglomerate features, which may be due to excessive nucleation of GPTS onto the Si–OH surface.

**Table 2:** Dynamic Contact Angle and Surface Free Energy of Si-GPTS and Si-GPTS-APTES-SBA

sample	contact angle (H_2_O) (°)	contact angle (I_2_CH_2_) (°)	surface free energy (mJ m^–2^)
Si–OH	8.96 ± 1.6	n/a	n/a
Si-GPTS	56.90 ± 2.1	39.90 ± 0.5	48.6 ± 1.8
Si-GPTS-APTES-SBA	27.63 ± 3.9	26.23 ± 5.3	66.70 ± 5.8

**Figure 5:**
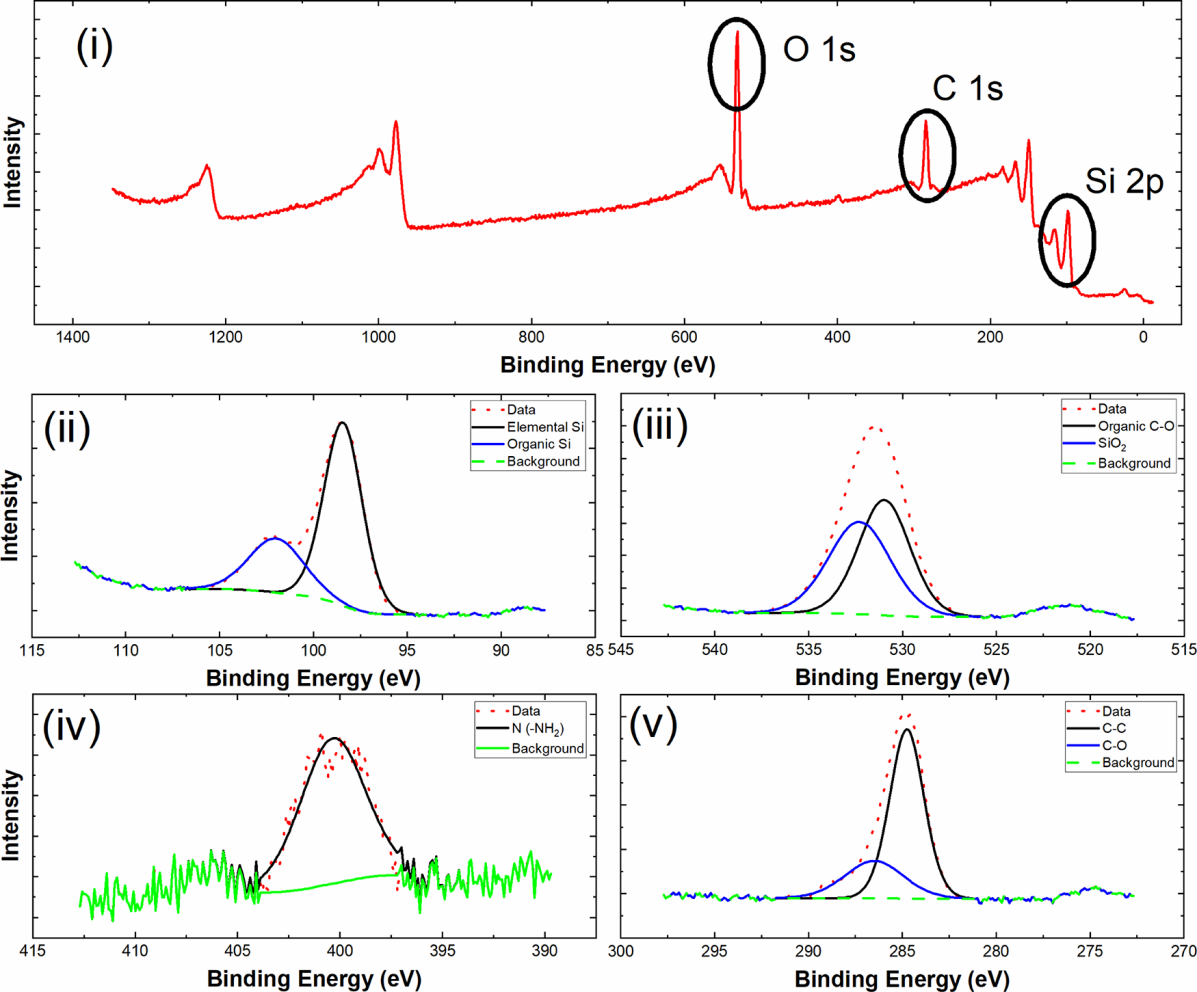
XPS survey spectra of (i) Si-GPTS-APTES-SBA with binding energies of (ii) Si 2p, (iii) O 1s, (iv) N 1s, and (v) C 1s scans.

X-ray photoelectron spectroscopy (XPS) analysis was used to examine the surface chemical properties of the Si-GPTS-APTES-SBA surface (Figure 5i) while also examining the surface composition and make-up of the core-level binding energies of Si 2p, O 1s, N 1s, and C 1s. The XPS spectrum (Figure 5ii) showed characteristic organic and elemental silica peaks at 103 and 97.5 eV, respectively. The O 1s scan (Figure 5iii) shows peaks at 532 and 529 eV, which are attributed to Si–O_2_ and organic C–O, respectively.^27^ In addition, binding energies typical of electrons from the N 1s chemical species were counted (Figure 5iv) due to the presence of amine groups in APTES with a binding energy peak at approximately 400 eV.^17^ Furthermore, from the XPS survey, the presence of an amino group peak indicates that the attachment of SBA-APTES to the surface of Si-GPTS has occurred as this nitrogen peak was absent from the Si-GPTS scan, confirming that SBA-APTES has attached to the surface. The C 1s scan (Figure 5v) also revealed peaks at 284 eV from C–H and C–C of the alkyl group of GPTS and from adventitious hydrocarbon contamination, while the peak at 286.6 eV was from C–O–C and the oxirane ring of GPTS. SEM analysis of Si-GPTS-APTES-SBA surfaces is shown in Figure 4iii,iv. These results strongly indicate that SBA-APTES was bound to the Si-GPTS surface. SEM analysis of Si-GPTS-APTES-SBA showed that no multilayer agglomerates were formed but instead isolated “specks”. Overall, these results indicate that SBA-APTES has been attached to the functionalized Si-GPTS-modified surface.

Piranha treatment of Si substrates is widely known to increase the density of free hydroxy (−OH) groups, facilitating the functionalization of the Si surface through silanization with the methoxy groups of GPTS *via* a hydrolysis reaction mechanism as outlined elsewhere.^17,26^ Then, the irreversible attachment of SBA-APTES to Si-GPTS occurs *via* a nucleophilic epoxide ring opening reaction between the amine groups from APTES and the oxirane ring from GPTS. This results in the covalent grafting of SBA-APTES to Si-GPTS.^28,29^ By directly attaching the support materials on the packaging substrate, this could facilitate the targeted release of the OEO antimicrobial directly onto the food surface while protecting the naturally volatile oil from the food matrix and packaging material.

### 2.3 OEO Loading and Assessment of the Antimicrobial Activity of Bare SBA-15 and SBA-APTES

Oregano essential oil (OEO) was loaded into either bare SBA-15 or SBA-APTES *via* absorption of the EO into the support material or through a drop-cast method onto the fabricated Si-GPTS-SBA-APTES surfaces. The loading of EO into SBA was confirmed through elemental analysis and FTIR. The antimicrobial activity of SBA-OEO (bare SBA-15 loaded with OEO) and SBA-APTES-OEO (SBA-APTES loaded with OEO) was measured by determining the minimum inhibition concentration (MIC) against the target microorganism. The antimicrobial activity of Si-GPTS-SBA-APTES-OEO was assessed using a disk diffusion assay.

The C, H, and N elemental analysis of SBA-15 and SBA-OEO showed that after OEO was loaded into the SBA-15 support material, an increase in the amount of elemental C, H, and N was observed from the secondary metabolites that make up OEO (Table 3). Moreover, the weight of bare SBA and SBA-APTES was taken before and after being loaded with OEO, with results showing increased weight by 65 and 63%, respectively. The results indicated that SBA and SBA-APTES can be used as support materials to successfully absorb OEO.

**Table 3:** Elemental Analysis of C, H, and N before OEO Loading of SBA-15 and after OEO Loading

element	SBA-15	SBA-OEO
C	Nil	29.63
H	0.74	3.99
N	Nil	Nil

FTIR spectra also confirmed successful loading of OEO into SBA (Figure 3ii). The FTIR spectra of pure OEO showed sharp characteristic peaks at 2959 cm^–1^ (−CH stretching), 1589 cm^–1^ (N–H bending), 1458 cm^–1^ (−CH_2_ bending), 1253 cm^–1^ (C–O–C stretching), 1117 cm^–1^ (C–O–C stretching), and 937 cm^–1^ (C–H bending).^30^ Compared to SBA-15 (Figure 3i), the spectra of SBA-OEO and SBA-APTES-OEO showed the characteristic peaks of OEO, indicating that OEO was loaded into the support material (bare SBA-15 or SBA-APTES); however, apparently, no modification or interaction between the OEO and bare SBA-15 occurred.^30^

**Figure 6:**
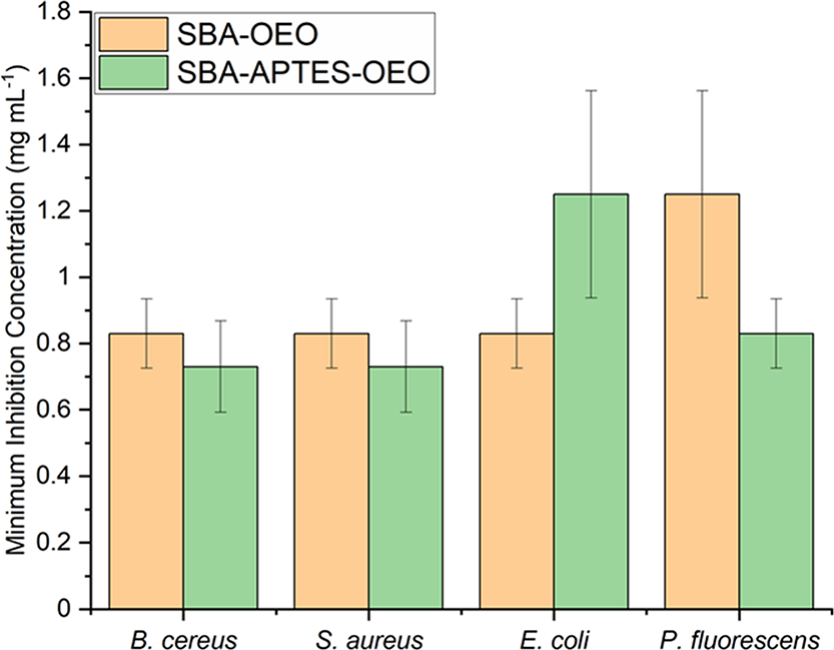
Minimum inhibition concentration of SBA-OEO (light orange bar) and SBA-APTES-OEO (light green bar) against *B. cereus*, *S. aureus*, *E. coli*, and *P. fluorescens*. Error bars represent the standard error of the mean of analysis of triplicate samples.

Upon OEO loading, the antimicrobial activity of SBA-OEO and SBA-APTES-OEO was assessed using an MIC assay and results showed that both SBA-OEO and SBA-APTES-OEO have good antimicrobial activity. Bare SBA-15 and SBA-APTES did not show any antimicrobial activity (see the Supporting Information). For SBA-OEO, concentrations of 0.83 and 1.25 mg mL^–1^ were required to inhibit Gram-negative *E. coli* and *P. fluorescens*, while a concentration of 0.83 mg mL^–1^ was required to inhibit the growth of both Gram-positive *S. aureus* and *B. cereus* (Figure 6). For SBA-APTES-OEO, a concentration of 0.83 mg mL^–1^ was required to inhibit the growth of both Gram-negative *E. coli* and *P. fluorescens*, while a concentration of 0.73 mg mL^–1^ was required to inhibit the growth of both Gram-positive *S. aureus* and *B. cereus* (Figure 6). The MIC values obtained from these experiments show an increase in antimicrobial efficacy compared to “unprotected” OEO. In a study carried out previously by our group, OEO was found to have MIC values of 8.3, 0.8, 8.8, and 3.8 mg mL^–1^ against *S. aureus*, *B. cereus*, *E. coli*, and *P. fluorescens*, respectively.^31^ It should be noted that the MIC assay indicates the lowest concentration of SBA-OEO or SBA-APTES-OEO that inhibits the growth of the targeted microorganism. However, it should be noted that this concentration does not indicate its bactericidal effect, which is the lowest concentration of an antibacterial agent required to kill bacteria over a fixed time. The concentration to achieve a bactericidal effect is typically greater than the reported MIC value.^32^ Statistical analysis of the results indicates no significant difference (*P* > 0.05) in the antimicrobial effect between using bare SBA-15 and SBA-APTES as support materials. However, SBA-APTES appeared to have better antimicrobial activity against Gram-positive *S. aureus* and *B. cereus* bacteria compared to bare SBA-15 when used as a support material for loading OEO. The greater antimicrobial activity of SBA-APTES-OEO against Gram-positive bacteria compared to SBA-OEO may be due to the fact that APTES altered the release profile of secondary metabolites of OEO, therefore effecting the interaction of OEO with the bacterial cell.^33^ It has also been reported that anchoring molecules with a positive charge on the surface of mesoporous silica particles can reduce microbial growth.^34^ The exact mechanism of the antimicrobial action of OEO is not fully understood; however, it is believed to be from a synergistic action between secondary metabolites such as *p*-cymene, thymol, and carvacrol found in OEO.^4^ In particular, carvacrol can disintegrate the outer membrane of Gram-negative bacteria, while in Gram-positive bacteria, the membrane permeability is altered, allowing permeation cations like H^+^ and K^+^.^4^ Antimicrobial action is further aided by *p*-cymene; although not inherently antimicrobial, it has a high affinity for bacterial cell membranes where it can substitute itself into the cell membrane, altering the physiological barrier properties, facilitating easier access for other more potent antimicrobial compounds.^35^ Moreover, results indicated that Gram-positive bacteria showed greater susceptibility to SBA-OEO and SBA-APTES-OEO compared to Gram-negative bacteria. Increased Gram-positive bacteria susceptibility to EO has been widely reported in the literature and is believed to be through the lipophilic ends on lipoteichoic acid in the cell membrane of Gram-positive bacteria, enabling the penetration of hydrophobic EO constituents into the internal cell structure.^36^ Conversely, the reduced susceptibility of Gram-negative bacteria was attributed to the role of extrinsic membrane proteins and cell wall lipopolysaccharides, limiting the diffusion of hydrophobic EO compounds into the microorganism.^36^

However, the disk diffusion assay on Si-GPTS-ATPES-SBA-OEO surfaces showed no antimicrobial activity (see the Supporting Information). This may perhaps be due to several factors such as the insufficient amount of SBA-APTES attached on the surface of Si-GPTS for the OEO to be absorbed into. For example, assuming that SBA was arranged in a spherical close-packed order and had an average diameter of 20 μm, the total number of SBA-15 units on the surface can be estimated to be 250,000 particles per 1 cm^2^. Then, the volume of SBA-15 can be calculated using 4/3π*r*^3^ to be 1.046 μL. The average load ability of the SBA was worked out from the weight before and after OEO loading and was found to be approximately 35%. Therefore, we can assume that the maximum volume of OEO on a 1 cm^2^ surface was 0.336 μL. Using the maximum MIC of 1.25 mg mL^–1^, we can determine that the volume of OEO needed for MIC was 0.26 μL. However, using ImageJ to estimate the SBA surface coverage from the SEM analysis (Figure 4iii,iv) showed that coverage was approximately 10%, which is therefore well below the required volume to show an antimicrobial effect. In addition, the interaction of APTES with the GPTS surface may perhaps reduce the number of available pores for adsorption of the OEO, therefore “blocking” OEO uptake into the mesospheric support material.

## 3 Conclusions

In this work, we present a novel approach to attach SBA-APTES to a GPTS-modified surface and have demonstrated that bare SBA-15 and SBA-APTES are effective support materials for loading OEO. The modification of bare SBA-15 with APTES did not negatively impact the antimicrobial activity of OEO against common food spoilage microorganisms *E. coli*, *B. cereus*, *S. aureus*, and *P. fluorescens*. Given the strong antimicrobial activity and GRAS nature of SBA-OEO and SBA-APTES-OEO, they could potentially be applied *via* a simple “sprinkle” method (like salt) directly on a food product as a route of investigation. Moreover, due to the strong absorption properties of SBA materials, other EOs such as thyme, rosemary, etc., can be used as a flavor delivering system. However, toxicity and release kinetics studies of these materials need to be carried out. Nonetheless, this work has shown that the functionalization of the SBA support material with an amine-terminated molecule does not significantly impact the antimicrobial activity of the EO. However, to enhance the antimicrobial properties of the developed surfaces, further studies such as the use of other methods of loading EO or perhaps through the use mesoporous silica nanoparticles or the insertion of SBA-OEO on edible films needs to be performed, in addition to the developed Si-GPTS-ATPES-SBA-OEO surfaces included in this study.

## 4 Materials and Methods

### 4.1 Materials

Oregano essential oil (OEO) was purchased from Lionel Hitchens Ltd. (Barton Stacey, Hampshire, UK). Mueller-Hinton Broth (MHB), Mueller-Hinton Agar (MHA), and Maximum Recovery Diluent (MRD) were purchased from Oxoid (Basingstoke, UK). *Escherichia coli* (*E. coli*) (NCIMB 11943), *Bacillus cereus* (*B. cereus*) (NCIMB 9373), *Staphylococcus aureus* (*S. aureus*) (NCIMB 13062), and *Pseudomonas fluorescens* (*P. fluorescens*) (NCIMB 9046) were maintained on Tryptic Soy Agar slants until use at 4 °C. The siliceous SBA-15 mesoporous material was purchased from Glantreo, Ireland. Blanket Si substrates were purchased from Sil’tronix, France. Sulfuric acid (ACS reagent, 95–98%), 3-aminopropyltriethoxysilane (99%) (APTES), 3-glycidyloxypropyltrimethoxysilane (>98%) (GPTS), hydrogen peroxide solution (30%), 2-propanol (CHROMASOLV, for high-performance liquid chromatography (HPLC), 99.9%), and dimethyl sulfoxide (DMSO) (anhydrous 99.9%) were all purchased from Sigma-Aldrich, Ireland. Deionized water was purchased from Acros Organics and was used as necessary.

### 4.2 Functionalization of SBA-15 with APTES

APTES was grafted to the surface of bare SBA-15 using DMSO as a novel solvent for this process. Briefly, 1 g of bare SBA-15 was placed into a 50 mL flask with a magnetic stirrer bar to which 20 mL of DMSO was added. To the SBA-DMSO solution, 2 mL of APTES was added dropwise and was allowed to react for 20 h before being washed with 20 mL of DMSO and 2-propanol and finally washed with three aliquots of 20 mL of deionized water. The SBA-15 grafted with APTES (SBA-APTES) was then dried for 1 h in a vacuum oven at 90 °C.

### 4.3 Preparation of the GPTS-Modified Si Surface

Si wafers were prepared for GPTS attachment by cutting Si into 1 cm^2^ wafers before being placed into a round-bottom flask with 40 mL of piranha solution (3:1 H_2_SO_4_:H_2_O_2_) for 1 h at 100 °C and then placed in distilled water until use for up to an hour. GPTS was attached to the hydroxylated Si by immersing the wafers into 9% (v/v) GPTS in DMSO solution and reacted for 20 h at 90 °C. The grafted wafers were removed, washed, and sonicated in DMSO, 2-propanol, and deionized water, dried using N_2_ gas, and placed in sample holders until further use.

### 4.4 Attachment of Si-GPTS with SBA-APTES

The SBA-APTES was attached to the Si-GTPS surface as outlined in the graphical abstract. Briefly, Si-GPTS wafers were submerged in 25 mL of deionized H_2_O to which 1 g of SBA-ATPES was added and the solution was then stirred for 2 h at 90 °C before removal. To remove unbound SBA-APTES, Si-GPTS-APTES-SBA was washed with deionized water and dried under a stream of N_2_ gas.

### 4.5 Loading of Oregano Essential Oil into SBA, SBA-APTES, and Si-GPTS-APTES-SBA

#### 4.5.1 Loading into Bare SBA-15 and SBA-ATPES

Before loading OEO into bare SBA-15 and SBA-APTES, OEO was dissolved in absolute ethanol to make a 10% (v/v) solution (as high concentrations of oil were found to be too viscous for effective loading). To this ethanolic solution, 0.5 g of either SBA-15 or SBA-APTES was added and then allowed to dry for 72 h at room temperature (21 °C) in a partially closed container to ensure full evaporation of the solvent.

#### 4.5.2 Loading of Si-GPTS-APTES-SBA

Due to the process conditions, the OEO could only be loaded into SBA after it was grafted to the Si surface. Si-GPTS-APTES-SBA was loaded with 10% (v/v) OEO in ethanol solution by drop-casting directly onto the wafer surface. Once the solution had attached onto the wafer, the OEO-loaded wafer was gently washed with sterilized deionized water to remove unabsorbed EO solution and allowed to dry before use in the modified disk diffusion assay.

### 4.6 Characterization

#### 4.6.1 Scanning Electron Microscopy (SEM) and Transmission Electron Microscopy (TEM)

Scanning electron microscopy (SEM) was carried out using a Karl Zeiss Ultra Plus field emission SEM with a Gemini column. The samples were placed on carbon tape and then adhered to a stainless-steel stub before being placed in the instrument’s chamber. It was operated at 5 keV and various magnifications were used as required. Transmission electron microscopy (TEM) was carried out using a JOEL 2100 at an operating voltage of 200 kV. The images were acquired in bright field mode.

#### 4.6.2 Fourier Transform Infrared Spectroscopy (FTIR)

Fourier transform infrared spectroscopy (FTIR) analysis of OEO, APTES, SBA-15, SBA-OEO, and SBA-ATPS-OEO was performed on a Varian 660-IR spectrometer (Varian Resolutions, Varian Inc., Victoria, Australia) using a diamond crystal ATR Golden Gate (Specac). Data were taken as the average of 32 scans at 2 cm^–1^ resolution in a wavenumber range from 4000 to 500 cm^–1^.

#### 4.6.3 X-ray Photoemission Spectroscopy (XPS)

X-ray photoelectron spectroscopy was performed under ultrahigh vacuum conditions (<5 × 10^–10^ mbar) on a VG Scientific ESCAlab Mk II system equipped with a hemispherical analyzer using Al Kα X-rays (1486.6 eV). The emitted photoelectrons were collected at a take-off angle of 90° from the disks’ surface. The analyzer pass energy for the survey scans was 200 eV. The binding energy scale was referenced to the adventitious carbon 1s core-level scans at 284.8 eV. Core-level scans of Si 2s, C 1s, N 1s, and O 1s were examined.

#### 4.6.4 N_2_ Adsorption–Desorption Isotherms

The surface area, pore diameter, pore volume, and pore size distribution measurements of the samples were performed based on the sorption technique using the Micromeritics Tristar II surface area analyzer (Micrometrics, Norcross, GA, USA). The specific surface area of the samples was calculated using the multipoint Brunauer, Emmett, and Teller (*S*_BET_) method in the relative pressure range *P*/*P*_0_ = 0.05–0.3. The specific pore volume, pore diameter, and pore size distribution curves were computed based on the Barrett–Joyner–Halenda (BJH) method. The sorption analysis was carried out at 77 K and each sample was degassed under nitrogen for 5 hours at 200 °C prior to analysis.

#### 4.6.5 Elemental Analysis

Elemental analysis was carried out on SBA-15 and SBA-OEO to determine the percentages of carbon, nitrogen, and hydrogen in the sample. The analysis was performed on an Exeter Analytical CE 440 elemental analyzer. All samples analyzed were carried out in triplicate.

#### 4.6.6 Thermogravimetric Analysis (TGA–DTG)

To evaluate the influence of temperature on the adsorbent stability, the adsorbents were studied by thermogravimetric analysis. All TG/first DTG curves were obtained on a Model TGA 2950 high-resolution thermogravimetric analyzer V5.4a on a temperature level from 30 to 800 °C with a warming speed of 5 °C min^–1^ under nitrogen flow.

#### 4.6.7 Dynamic Contact Angle (DCA)

Dynamic contact angle (DCA) and surface free energy were calculated from the advancing and receding water contact angles and were recorded on three different regions of each sample as outlined by Lundy et al.^37^ Briefly, 60 nL of the liquid was dispensed on the material surface at a flow rate of 5 nL s^–1^ using a microinjection syringe pump (SMARTouch, World Precision Instruments, Sarasota, FL, USA) with a needle (ϕ = 130 μm), and images were captured with a monochrome industrial camera (DMK 27AUR0135, The Imaging Source, Bremen, Germany). Contact angles were calculated using a piecewise polynomial fit (ImageJ, ver. 1.46, DropSnake plugin). The same procedure was used to determine diiodomethane (CH_2_I_2_) contact angles. The surface free energy values were calculated from contact angles of deionized water and diiodomethane using the Owens–Wendt model (eq 1.).
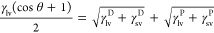
1

Surface energy values of H_2_O (*γ*_lv_^D^/*γ*_lv_^P^ = 21.8/50.8 mJ m^–2^) and CH_2_I_2_ (*γ*_lv_^D^/*γ*_lv_^P^ = 48.5/2.3 mJ m^–2^) were used.

#### 4.6.8 Atomic Force Microscopy (AFM)

Atomic force microscopy (AFM, Park Systems, XE-7, South Korea) measurements on Si-GPTS were performed in noncontact mode with high-resolution, silicon microcantilever tips. Topographic images were recorded at a resonance frequency of 270–300 kHz.

#### 4.6.9 Antimicrobial Assay

The antimicrobial activity of SBA-OEO and SBA-APTES-OEO against Gram-positive bacteria *S. aureus* and *B. cereus* and Gram-negative bacteria *E. coli* and *P. fluorescens* was assessed. Before use, all pure culture bacteria were grown for 18 h at 30 °C (*P. fluorescens* and *B. cereus*) or 37 °C (*S. aureus* and *E. coli*) in Mueller-Hinton Broth (MHB) (Oxoid, UK) under constant agitation at 170 rpm on an orbital shaker (Innova 2300, New Brunswick, Germany). These cultures were then used to determine the following.

##### 4.6.9.1 Minimum Inhibition Concentration (MIC) Assay

The antimicrobial activity of SBA and SBA-APTES loaded with OEO was measured by determining the minimum inhibitory concentration (MIC) against the target microorganisms in 96-well flat-bottom tissue culture microplates (Sarstedt Inc., NC, USA) according to the NCCLS (2000) broth microdilution method as described by Cruz-Romero et al.^38^ Bacterial strains were cultured overnight, at the appropriate temperature, adjusted to a final density of 10^5^ CFU/mL using Maximum Recovery Diluent, and used as an inoculum within 15 min of preparation as outlined previously by Sullivan et al.^39^ Briefly, 100 μL of double-strength MHB (2XMHB) was added to each well in rows A to F, 200 μL of adjusted bacterial culture suspension was added to row H in columns 1–11, and 200 μL of sterile 2XMHB was added to column 12. In each well of row G, 150 μL of SBA-OEO or SBA-APTES-OEO was dispensed in sterile distilled water and a threefold serial dilution was performed by transferring 50 μL of antimicrobial solutions from row G into the corresponding wells of row F through row B. After mixing, 50 μL of the resultant mixture on row B was discarded. Finally, using a 12-channel electronic pipette (Model EDP3-Plus, Rainin, USA), 15 μL of the tested microorganisms was pipetted from each well in row H into the corresponding wells in row A followed by rows B to G. Positive (row A) and negative growth controls (column 12) were included in each assay plate. The inoculated plates were incubated in a wet chamber for 24 h at 30 °C (*P. fluorescens* and *B. cereus*) or 37 °C (*E. coli* and *S. aureus*). The lowest concentration showing the inhibition of growth was considered to be the MIC for the target microorganisms. The test was repeated in triplicate.

##### 4.6.9.2 Modified Disk Diffusion Assay

The antimicrobial activity of SBA-functionalized surfaces containing OEO was also assessed using a modified agar diffusion method. MHA plates were swabbed with the target microorganism grown overnight at the appropriate temperature and adjusted to a final density of ∼10^5^ CFU mL^–1^. SBA-functionalized surface substrates containing OEO were then placed in the middle of the inoculated agar plates and incubated for 24 h at 30 °C (*P. fluorescens* and *B. cereus*) or 37 °C (*S. aureus* and *E. coli*). A streptomycin antibiotic disc (10 μg) was used as the positive control, while unloaded SBA-functionalized surfaces without EO were used as the negative control. The inhibition zone around the substrate indicated the antimicrobial activity against the target microorganism. The inhibition zone (in millimeters) was measured using an electronic caliper (Model ECA 015D Moore &Wright, Paintain Tools Ltd., Birmingham, UK).

### 4.7 Statistical Analysis

Data for antimicrobial tests were analyzed for means, standard deviations, and analysis of variance. One-way analysis of variance of data was carried out using the SPSS 24 for Windows (SPSS statistical software, IBM Corp., Armonk, NY, USA) software package. Differences between pairs of means were resolved by means of confidence intervals using Tukey’s test; the level of significance was set at *P* < 0.05.

## Supporting Information Available

FTIR spectra of SBA, APTES, and OEO and example photograph of MIC assay results (PDF)

## Abbreviations Used

APTES: (3-aminopropyl)triethoxysilane
GPTS: 3-glycidyloxypropyltrimethoxysilane
AFM: atomic force microscopy
*B. cereus*: *Bacillus cereus*
*D*_BJH_: Barrett–Joyner–Halenda pore size
*S*_BET_: Brunauer, Emmett, and Teller surface area
DMSO: dimethyl sulfoxide
*E. coli*: *Escherichia coli*
EO: essential oil
FDA: Food and Drug Administration
FFT: fast Fourier transform
FTIR: Fourier transform infrared spectroscopy
MRD: Maximum Recovery Diluent
MIC: minimum inhibition concentration
MCM-41: Mobil Composition of Matter No. 41
MHA: Mueller-Hinton Agar
MHB: Mueller-Hinton Broth
NAM: natural antimicrobial material
OEO: oregano essential oil
*P. fluorescens*: *Pseudomonas fluorescens*
SBA-15: Santa Barbara Amorphous 15
SEM: scanning electron microscopy
*S. aureus*: *Staphylococcus aureus*
TGA: thermogravimetric analysis
*V*_total_: total pore volume
TEM: transmission electron microscopy
XPS: X-ray photoelectron spectroscopy
