# Supplementary material for: The Use of Porous Silica Particles as Carriers for a Smart Delivery of Antimicrobial Essential Oils in Food Applications
Source: ACS Omega. 2021 Nov 3;6(45):30376–85. doi: 10.1021/acsomega.1c03549 (PMC8603183; doi:10.1021/acsomega.1c03549)
Supplement: Supplementary file 1 — ao1c03549_si_001.pdf [file ao1c03549_si_001.pdf]

The use of porous silica particles as carriers for a smart delivery of antimicrobial essential oils in food applications

David J. Sullivan, [a,#] Tom F. O'Mahony [a], Malco C. Cruz-Romero [b*], Enda Cummins [c], Joseph P. Kerry [b], Michael A. Morris [a*]

[a] AMBER Research Centre and the School of Chemistry, Trinity College Dublin, Dublin 2, Ireland

[b] Food Packaging Group, School of Food & Nutritional Sciences, University College Cork, Cork, Ireland

[c] UCD School of Biosystems and Food Engineering, Agriculture and Food Science Centre, University College Dublin, Belfield, Dublin 4, Ireland.

Footnote #; Current institution AMBER Research Centre and the School of Chemistry, Trinity College Dublin, Dublin 2, Ireland; however, this work was carried out in the School of Chemistry, University College Cork, Cork, Ireland.

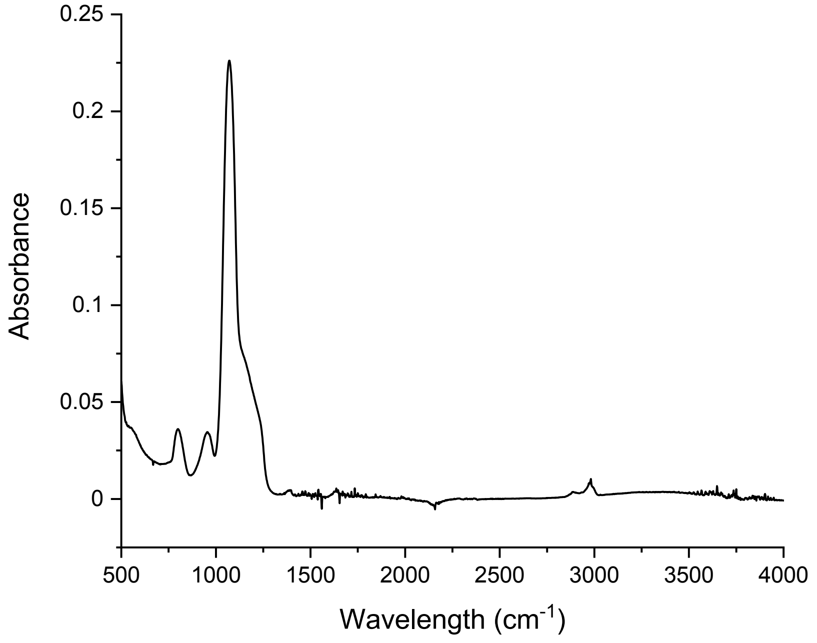

Figure S1. FTIR spectrum of bare SBA-15

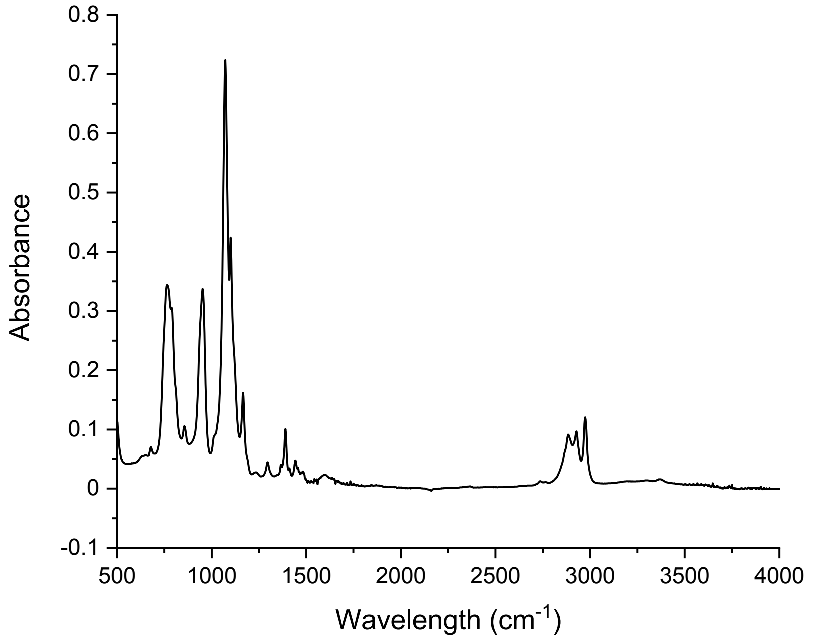

Figure S2. FTIR spectrum of APTES

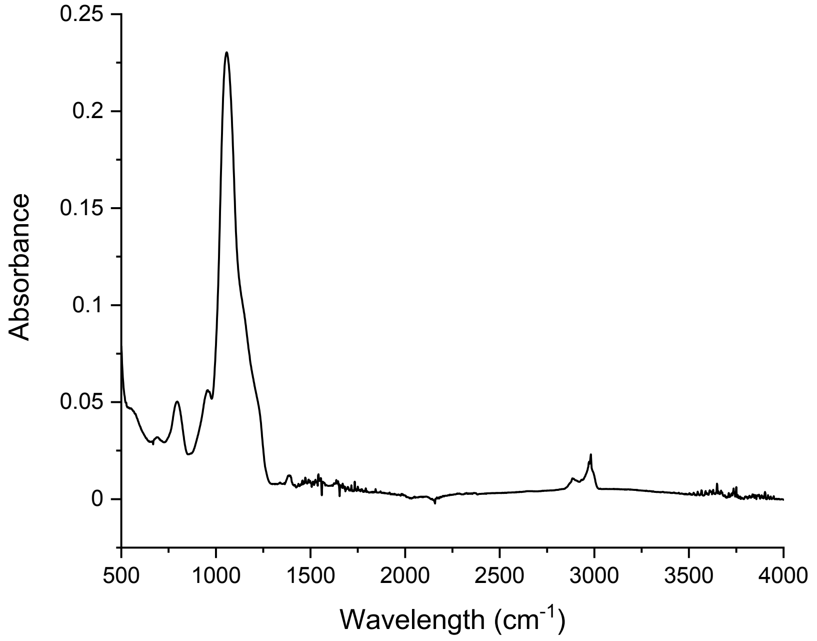

Figure S3. FTIR spectrum of SBA-APTES

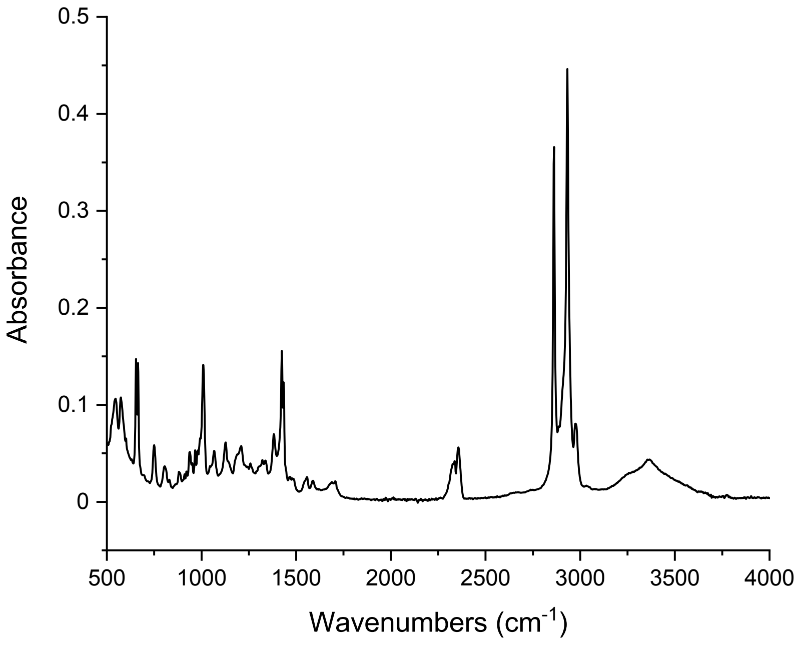

Figure S4. FTIR spectrum of OEO

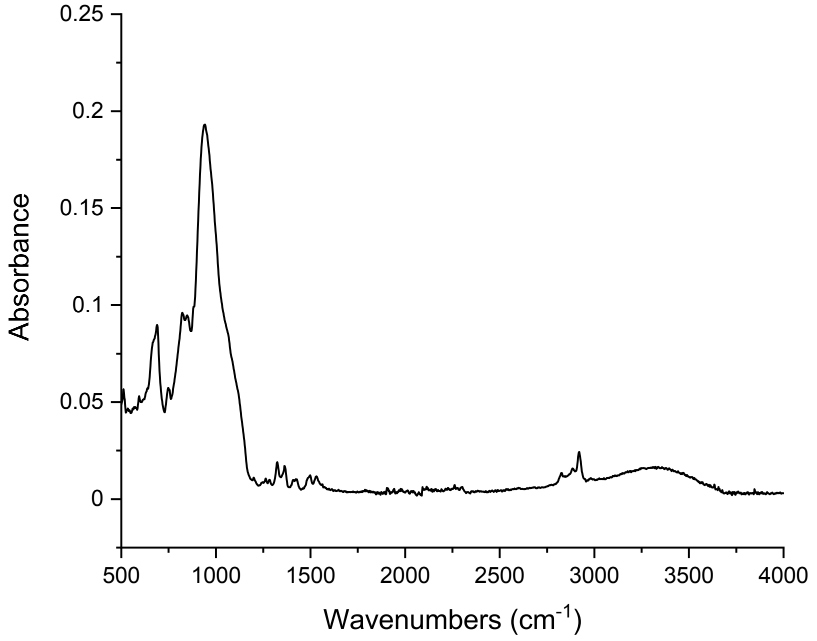

Figure S5. FTIR spectrum of SBA-OEO

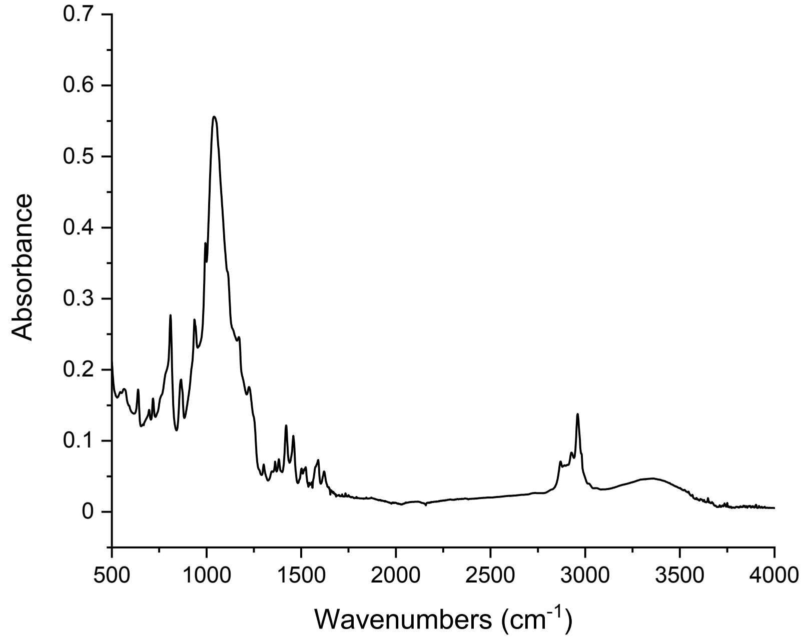

Figure S6. FTIR spectrum of SBA-ATPES-OEO

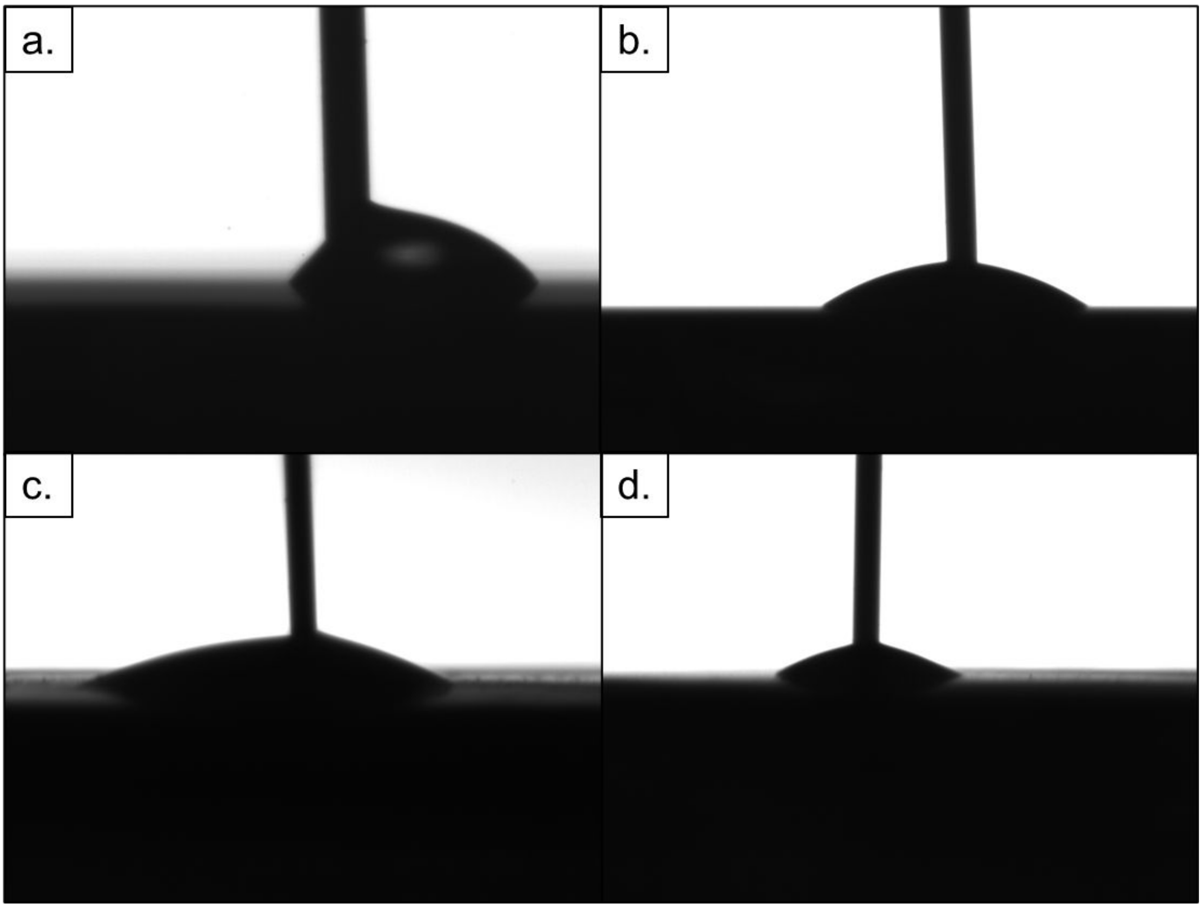

Figure S7. Dynamic contact images of Si-GPTS using H_2O (a.), and CH_2I_2 (b.) and Si-GPTS-APTES-SBA using H_2O (c.), and CH_2I_2 (d.)

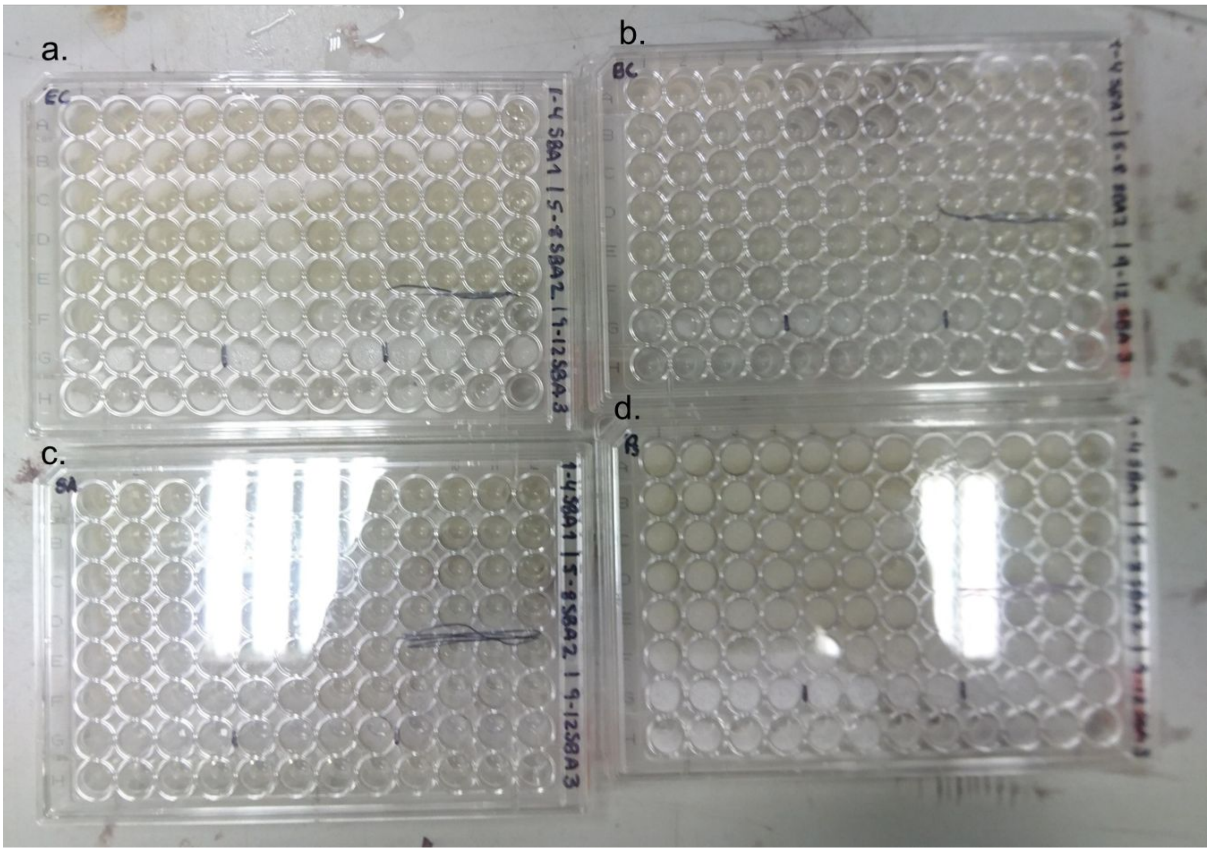

Figure S8. MIC assay of SBA-15 (column 1-4), SBA-APTES (column 5-8), and SBA-OEO (column 8-11) against a.) E. coli, b.) B. cereus, c.) S. aureus, and d.) P. fluorescens. Column 12 is the negative control and row A is the positive control. Results show that turbidity (i.e., microbial growth) is present for column 1 – 8 which contain bare SBA-15 and SBA-APTES, Column 9 -11 contain SBA-OEO where the no turbidity was observed in row E and F. ***Photograph courtesy of David J. Sullivan. Copyright 2021.***
